# *tert*-Butylphenolic Derivatives from *Paenibacillus odorifer*—A Case of Bioconversion

**DOI:** 10.3390/molecules23081951

**Published:** 2018-08-05

**Authors:** Thi-Bach-Le Nguyen, Olivier Delalande, Isabelle Rouaud, Solenn Ferron, Laura Chaillot, Rémy Pedeux, Sophie Tomasi

**Affiliations:** 1 University of Rennes 1, CNRS, ISCR—UMR 6226, F-35000 Rennes, France; nguyen.bachle@yahoo.com (T.-B.-L.N.); isabelle.rouaud@univ-rennes1.fr (I.R.); solenn.ferron@univ-rennes1.fr (S.F.); 2 University of Rennes 1, CNRS, IGDR—UMR 6290, F-35000 Rennes, France; olivier.delalande@univ-rennes1.fr; 3 Chemistry, Oncogenesis, Stress, Signaling, Centre Eugène Marquis, Université de Rennes 1, INSERM U1242, 35000 Rennes, France; laura.chaillot@univ-rennes1.fr (L.C.); remy.pedeux@univ-rennes1.fr (R.P.)

**Keywords:** *tert*-butylphenols, *Paenibacillus odorifer*, *Rhizocarpon geographicum*, bioconversion

## Abstract

Two compounds (**1**) and (**2**) containing *tert*-butylphenol groups were, for the first time, produced during the culture of *Paenibacillus odorifer*, a bacterial strain associated with the crustose lichen, *Rhizocarpon geographicum*. Their entire structures were identified by one-dimensional (1D) and two-dimensional (2D) NMR and high-resolution electrospray ionisation mass spectrometry (HRESIMS) spectroscopic analyses. Among them, Compound **1** exhibited significant cytotoxicity against B16 murine melanoma and HaCaT human keratinocyte cell lines with micromolar half maximal inhibitory concentration (IC_50_) values. Furthermore, after supplementation studies, a putative biosynthesis pathway was proposed for Compound **1** throughout a bioconversion by this bacterial strain of butylated hydroxyanisole (BHA), an antioxidant polymer additive.

## 1. Introduction

**Figure 1:**
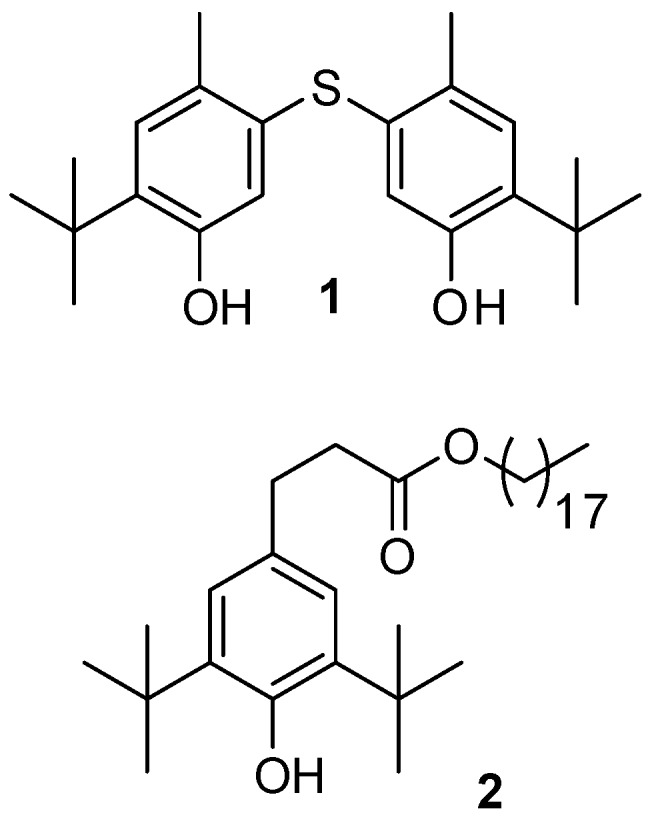
The structure of *tert*-butylphenolic derivatives found in the culture of *Paenibacillus odorifer.*

Organic products with branched *tert*-butyl groups represent a relatively important number of active compounds [1,2]. In the past, this group was already exploited in many organic syntheses due to its chemical reactivity. There are more than 200 compounds containing *tert*-butyl groups, described as natural products with interesting bioactivities [3]. Indeed, the *tert*-butyl moiety can be found in a variety of compounds produced by various sources, such as plants, fungi, algae, cyanobacteria [4,5,6,7,8,9], and especially from bacteria which were admitted as a source of novel and interesting bioactive products [10]. The *tert*-butylated compounds from natural sources reported in these papers often belong to classes of terpenes or polyketides. Although metabolites containing *tert-*butyl functionality on the aromatic ring are considered to be rare, several *tert*-butylphenyl derivatives were already isolated from organisms and they also exhibited remarkable antitumor, antibacterial, and antioxidant activities [11,12,13]. Chemical studies on *Paenibacillus odorifer*, a bacterium associated with the crustose lichen, *Rhizocarpon geographicum,* led to the identification of two *tert*-butylphenol derivatives (**1**, **2**) described in Figure 1. Interestingly, aromatic compounds simultaneously bearing a *tert*-butyl group and a sulfur atom are really uncommon in nature. The aims of this work were to characterize these compounds, to give some hypothesis about their origin, and to evaluate their cytotoxic activities. Herein, to our knowledge, is the first report of the bacterial production of Compound **1** carrying a *tert*-butyl phenol moiety and a sulfur atom.

## 2. Results

### 2.1. Process of Purification

*Paenibacillus odorifer* (*P. odorifer*) was cultivated via a scale-up (4.0 L) shaken fermentation at 25 °C, pH 7, 120 rpm with 1% (*v*/*v*) inoculum in GYM *Streptomyces* medium supplemented with CaCO_3_. From the broth, the excreted metabolites were collected using Amberlite^®^ XAD-7-HP resin, and the resulting extract was separated via a combination of liquid/liquid extraction and normal-phase flash chromatography to give several fractions. Final purification combining silica gel chromatography, semi-preparative HPLC, and preparative silica thin-layer chromatography (TLC) afforded 5,5′-thiobis(2-*tert*-butyl-4-methylphenol) (**1**; 5.0 mg) and octadecyl 3-(3,5-di-*tert*-butyl-4-hydroxyphenyl)propanoate (**2**; 5.9 mg).

### 2.2. Structural Elucidation

**Figure 2:**
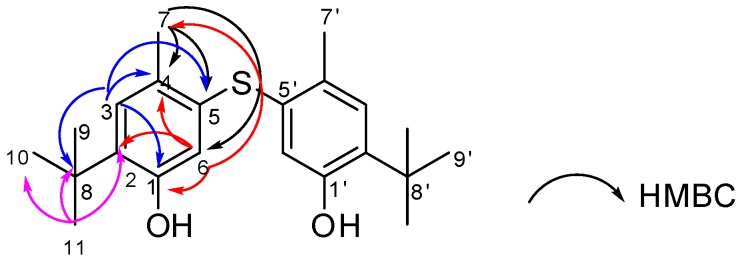
Key correlations for the structural assignment of **1**.

**Figure 3:**
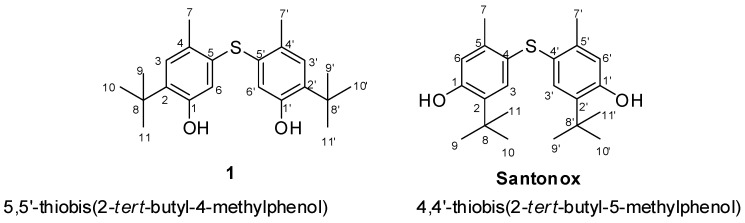
Structures of Compound **1** and Santonox.

Compound **1** was obtained as a white amorphous powder. Its molecular formula was assigned as C_22_H_30_O_2_S from the [M − H]^−^ ion at *m/z* 357.1983 according to high-resolution electrospray ionisation mass spectrometry (HRESIMS) data, and required eight degrees of unsaturation. Its ^13^C-NMR data highlighted nine carbon signals, which were classified by Jmod and HSQC analyses as two aromatic methine carbons, four aromatic quaternary carbons, one aliphatic quaternary carbon, and four methyl carbons (Table 1). The lack of coupling between two aromatic protons at δ_H_ 6.56 ppm (1H, s, H-6) and 7.00 (1H, s, H-3) indicated a *para*-relationship between them, and the existence of a 1,2,4,5-tetrasubstituted phenyl nucleus. The presence of three methyl groups at δ_H_ 1.28 (9H, s, H-9/H-10/H-11) along with their HMBC correlations to a quaternary carbon at δ_C_ 34.4 (C-8) revealed the presence of a *tert*-butyl group which was one of the substituted groups on the aromatic ring. On the other hand, the ^13^C shift of C-2 at δ_C_ 134.6 together with its HMBC correlations with H-9/H-10/H-11 confirmed the position of the *tert*-butyl group at C-2. In addition, analysis of the HMBC spectrum exhibiting the correlations from H-3 to C-8 (δ_C_ 34.4), C-4 (δ_C_ 125.5), C-5 (δ_C_ 137.8), and C-1 (δ_C_ 153.4) provided the presence of a methyl group at C-4, a hydroxyl group at C-1, and a sulfur-bond at C-5. The latter could also be indicated through the HMBC correlations from the aromatic methyl protons at δ_H_ 2.28 (3H, s, H-7) to C-4, C-5, and C-6 (δ_C_ 118.8). Moreover, HMBC correlations from H-6 to C-1, C-2, C-4, and C-7 were also observed. Interestingly, NOE correlations between two kinds of protons at δ_H_ 7.00 (H-3) and δ_H_ 1.28 (H-9/H-10/H-11) indicated that the *tert*-butyl group possessed a position close to C-3. The remaining structural assignment of Compound **1** (Figure 2) required C_11_H_15_O, which corresponds to the already assigned part of Compound **1**. On the basis of molecular formula and HMBC correlations, Compound **1** was suggested to possess a symmetrical structure containing two 1,2,4,5-tetrasubstituted phenyl nuclei which were linked via a sulfur bond This structure is close to that of a known synthetic compound Santonox (CAS registry number 96-69-5) 78 (Figure 3). This is the first natural isolation of Compound **1**.

From the comparison of the NMR spectroscopic data, minor differences in the chemical shifts of protons and carbons were highlighted for Compound **1** and standard Santonox. Thus, the identification of the exact structures could not be based on these data, with the exception of NOE correlations. For Santonox, the NOE correlations were clearly displayed between two series of protons at δ_H_ 7.00 (aromatic proton, H-3) and δ_H_ 1.28 (*tert*-butyl protons), and at δ_H_ 6.54 (aromatic proton, H-6) and δ_H_ 2.27 (methyl protons). For Compound **1**, the NOE experiments only highlighted the correlations between δ_H_ 7.00 (aromatic proton, H-3) and δ_H_ 1.28 (*tert*-butyl protons). The absence of the NOE correlation between H-6 (δ_H_ 6.56) and protons of the *tert*-butyl group (δ_H_ 1.28) indicated that H-6 in Compound **1** was not close to the *tert*-butyl group. NOE predictions obtained through molecular dynamics simulations confirmed the NOE experimental data (see Supplementary Materials, Figure S20 and Table S1). As a result, the data from NOE correlations finally highlighted that Compound **1** was an isomer of Santonox. Moreover, to our best knowledge, it is the first report of the isolation of Compound **1** from a bacterial culture.

**Figure 4:**
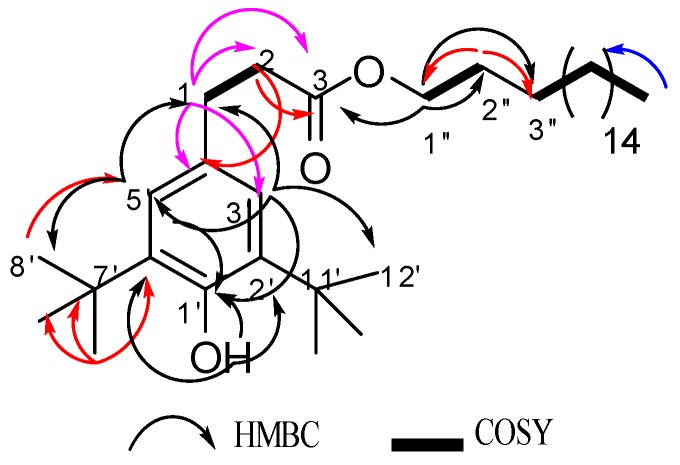
Key correlations for the structural assignment of **2**.

Compound **2** was isolated as a white solid and had a C_35_H_62_O_3_ molecular formula determined by the [M + Na]^+^ peak at *m/z* 553.4592 from its (+)HRESIMS data. The analysis of ^1^H-NMR and Jmod, along with HSQC data (Table 2), indicated the presence of two aromatic protons, 19 methylene groups (one of them oxygenated), seven methyl groups (six of them as singlets), four aromatic quaternary carbons, and one carbonyl carbon. The presence of two aromatic protons at δ_H_ 6.99 (2H, s, H-3′/H-5′) pointed to the existence of a 1,2,4,6-tetrasubstituted phenyl moiety (Figure 4). Four spin systems could be revealed via analysis of COSY correlations, corresponding to the C-1 to C-2, C-1″ to C-2″, C-3″ to C-17″, and C-17″ to C-18″ fragments. The HMBC correlations from an exchangeable proton (δ_H_ 5.07, 1H, bs) to C-1′ demonstrated that C-1′ might be substituted by a hydroxyl group, and it was confirmed by the ^13^C shift of C-1′ at δ_C_ 152.1. Compound **2** also presented two *tert*-butyl groups containing six methyl groups at δ_C/H_ 30.3/1.43 (18H, s, H-8′, H-9′, H-10′, H-12′, H-13′, H-14′) connected with two quaternary carbons at δ_C_ 34.3 (C-7′/C-11′) as two of the substituted groups on the aromatic ring. In addition, the HMBC data provided correlations from the protons of *tert*-butyl groups to C-7′/C-11′, C-2′/C-6′ (δ_C_ 135.8), C-3′/C-5′ (δ_C_ 124.8), and C-4′ (δ_C_ 131.1); and from H-3′/H-5′ to C-1′ (δ_C_ 152.1), C-3′/C-5′ (δ_C_ 124.8), and C-1 (δ_C_ 31.0). Moreover, the presence of two coupled methylene groups at δ_H_ 2.85 (2H, dd, *J* = 9.1, 6.9 Hz, H-1) and δ_H_ 2.60 (2H, dd, *J* = 9.1, 6.9 Hz, H-2) was also observed. The HMBC correlations from H-1 to C-4′, C-3′/C-5′, C-2 (δ_C_ 36.5), and C-3 (δ_C_ 173.4); and from H-2 to C-4′, C-1, and C-3 provided more evidence that this group made a linkage between a phenyl nucleus and a carbonyl carbon (Figure 4). Furthermore, the oxygenated methylene at δ_H_ 4.07 (2H, t, *J* = 6.8Hz, H-1″), δ_C_ 64.6 (C-1″) was linked to carbonyl carbon C-3 and to other several methylene groups as indicated by the HMBC data. Thus, the structure of Compound **2** was established as octadecyl 1-(2′,6′-di-*tert*-butyl-1′-hydroxyphenyl)propanoate, introduced in Figure 4. This structure was already reported in the literature [3]; however, its full NMR data are not yet published, and this is the first report of its isolation from a culture of *P. odorifer*.

### 2.3. Supplementation Assays 

**Figure 5:**
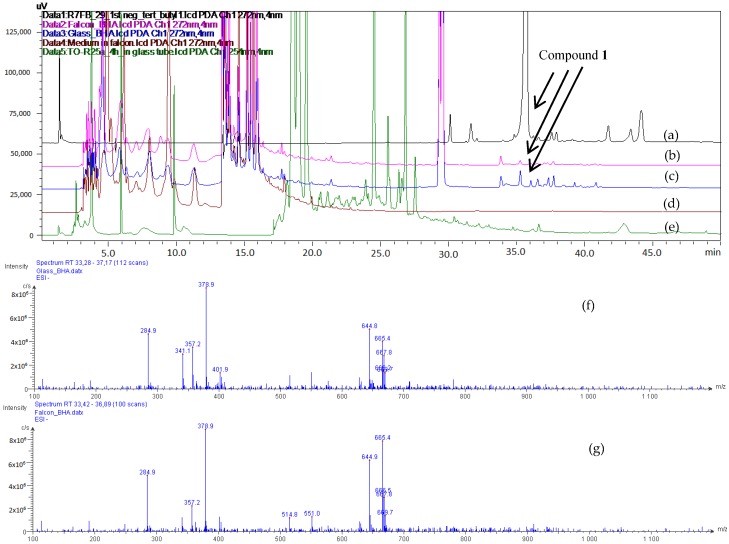
HPLC chromatograms of Compound **1** (**a**), of the extracts from *P. odorifer* culture supplemented with butylated hydroxyanisole (BHA) in the culture flask (**b**), or in the glass Erlenmeyer flask (**c**), of medium in the culture flask (**d**), and of the *Paenibacillus odorifer* (*P. odorifer*) culture in the Erlenmeyer flask (**e**). Electrospray ionisation (ESI)-MS (−) spectra of extracts from the *P odorifer *culture supplemented with BHA in the culture flask (**f**), or in the glass Erlenmeyer flask (**g**).

The structure of Compound **1** was determined to be closely related to that of butylated hydroxyanisole (BHA) which is approved as an antioxidant ingredient added to polymers, foods, and food-related products [14,15,16]. To respond to this issue, a supplementation of the culture of *P. odorifer* was carried out with standard BHA, put either in a culture flask (a kind of plastic vessel) or in an Erlenmeyer (a kind of glass vessel). Furthermore, the controls were based on the medium incubated in a culture flask and the culture of *P. odorifer* in an Erlenmeyer flask. The results from the LC–MS data are shown in Figure 5, and they highlighted that both extracts from the cultures supplemented with BHA in the culture flask and Erlenmeyer flask provided [M − H]^−^ ions at *m/z* 357 with a retention time of 35.7 min, which is characteristic of Compound **1**. However, Compound **1** could neither be found in the extract from the medium incubated in the culture flask, nor from the culture of *P. odorifer* in the Erlenmeyer flask.

**Figure 6:**
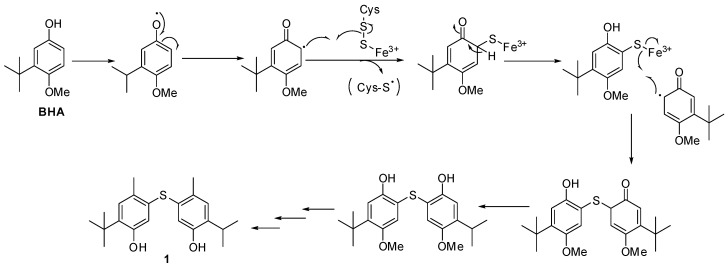
Putative biosynthetic pathway for Compound **1** from BHA supported by an iron–sulfur cluster protein with cysteine as a sulfur donor.

Additionally, the analysis of Fraction **1′,** which was a mixture of non-separable BHA and Compound **1**, partially purified from the culture supplemented with BHA in the Erlenmeyer flask, showed similar NOE correlations to Compound **1** between δ_H_ 6.99 and δ_H_ 1.28 (*tert*-butyl protons; see Supplementary Materials, Figure S13). Accordingly, we concluded that Compound **1** was converted by *P. odorifer* from BHA, which was detected in the medium incubated in the culture flask. Therefore, the biosynthetic pathway of Compound **1** is proposed in Figure 6, following the mechanism suggested by Fontecave [17,18] with some modifications. After an oxidative step of BHA, the formed phenoxy radical could react with cysteine as a sulfur donor to produce Compound **1** after further reactions. This reaction could be supported by an iron–sulfur cluster protein that was already reported in the genome of *P. odorifer* (gene symbol PODO_RS22860), described in the NCBI bank.

**Figure 7:**
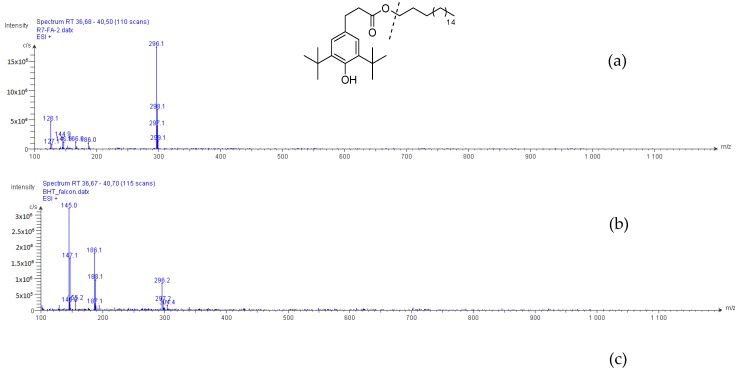

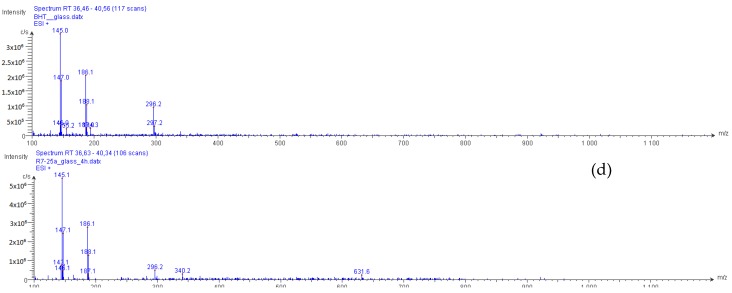
ESI-MS (+) spectra of Compound **2** (**a**), of the extracts from the culture supplemented with BHT in either the culture flask (**b**) or the Erlenmeyer flask (**c**), and of the culture of *P. odorifer* in the Erlenmeyer flask (**d**).

Compound **2**, as with Compound **1**, was isolated from the culture process using a culture flask in the pre-culture stage. In order to discover the origin of Compound **2**, butylated hydroxytoluene (BHT), with its close structure to that of Compound **2**, was used as a supplemented material during the culture of *P. odorifer*, put either in a culture flask or in an Erlenmeyer flask. The blank controls were the medium (without bacteria) in the culture flask and the culture of *P. odorifer* in the Erlenmeyer flask. The HPLC-MS data introduced in Figure 7 exhibited that Compound **2**, with a retention time at 38 min, was associated with an ion at *m/z* 296, which occurred in extracts from media supplemented with standard BHT in both the culture flask and the Erlenmeyer flask. This ion was detected in the MS spectrum of Compound **2** due to the hydrolysis of its ester group in LC–MS process. However, Compound **2** was not found in the medium put in the culture flask, but was found in the culture broth of *P. odorifer* in the Erlenmeyer flask. On the other hand, this compound was already reported in the literature [3] from Oakwood. Therefore, we propose that Compound **2** came from the bioconversion of BHT, or as a natural metabolite from the culture of *P. odorifer*. Furthermore, our *P. odorifer* strain could be considered as a new example of *tert*-butylphenol-utilizing bacterium.

### 2.4. Cytotoxic Activity

The biological activities of Compounds **1** and **2** were tested using a 3-(4,5-dimethylthiazol-2-yl)-2,5-diphenyltetrazolium bromide (MTT) assay on HaCaT (human keratinocytes) and B16 (murine melanoma) cell lines (Table 3) [19]. Although neither compound showed activity significantly greater than the positive control (doxorubicin) against the two cell lines, Compound **1** exhibited a significant half maximal inhibitory concentration (IC_50_) on B16 (4.75 µM) and HaCaT (8.38 µM), while Compound **2** was less active. Additionally, DNA damage assays, using γH2AX as a biomarker, were performed with Compound **1** on U2OS cells (Table 4). These cells are frequently used since they are sensitive to DNA damage. Although the compound was highly cytotoxic at 1 µM (cell death > 90%), no significant induction in γH2AX foci was observed at 1 µM or 0.1 µM within the nuclei, suggesting that no significant DNA damage was triggered compared to untreated cells. These results suggest that the cytotoxicity of Compound **1** was not driven by DNA damage.

## 3. Materials and Methods

### 3.1. General Experimental Procedures

One-dimensional (1D) and two-dimensional (2D) NMR spectroscopic data were recorded in MeOH-*d*_4_ and CDCl_3_ on a Bruker DMX 300 spectrometer (300 MHz (^1^H) and 75 MHz (^13^C), Bruker BioSpin, Billerica, MA, USA). NMR spectroscopic data were processed using the MestRenoVa version 10.0 software (Mestrelab Research, S.L., Santiago de Compostela, Spain). HRMS measurements for exact mass determination were performed with a Q-Extractive Focus at the Centre Regional de Mesure Physique de l’Ouest (CRMPO), Rennes, France. Analytical HPLC and semi-preparative HPLC were performed on a 5-µm Prevail C_18_ column (250 mm × 4.6 mm for the former, and 250 mm × 10 mm for the later), GRACE, Columbia, MD, USA.

### 3.2. Collection and Phylogenetic Analysis of PC-GYM-TO Strain

The PC-GYM-TO strain was isolated from the crustose lichen, *Rhizocarpon geographicum*, collected in Brittany, France in February 2015. The strain was identified at Banyuls/mer Platform (L. Intertaglia) as *Paenibacillus odorifer* based on 16S ribosomal RNA (rRNA) gene sequence analysis (GenBank accession number AJ223990). A comparative BLAST similarities search of the 16S rRNA gene sequence gave a 98.46% similarity to that of *P. odorifer* (Gene bank entry PODO_RS03805). After culture in GYM *Streptomyces* medium (containing 4 g of glucose (Sigma-Aldrich, St Louis, MO, USA), 4 g of yeast extract (Sigma-Aldrich, St Louis, MO, USA), 10 g of malt extract (Sigma-Aldrich, St Louis, MO, USA), 2 g of CaCO_3_ (Merck KGaA, St Frankfurter, Darmstadt, Germany), and 12 g of agar (Sigma-Aldrich, St Louis, MO, USA) in 1 L), the bacterium was stored in a mixture of 47.5% (*v*/*v*) glycerol, 47.5% (*v*/*v*) H_2_O, and 5% (*v*/*v*) DMSO at −80 °C with a reference of PC-GYM-TO (CORINT collection).

### 3.3. Cultivation and Extraction

*P. odorifer* (strain PC-GYM-TO) was cultured on GYM *Streptomyces* medium agar (2 g of glucose, 4 g of yeast extract, 4 g of malt extract, 2 g of CaCO_3_, and 12 g of agar in 1 L at pH 7). The inoculum was prepared by transferring one loop full of culture (PC-GYM-TO) from agar medium to a 250-mL culture flask, containing 50 mL of liquid GYM *Streptomyces* medium (2 g of glucose, 4 g of yeast extract, 4 g of malt extract, and 2 g of CaCO_3_ in 1 L at pH 7). The bacterium culture was grown at 25 °C on a rotary shaker incubator at 120 rpm for seven days. After seven days for pre-culture, 42 mL of bacterium culture was transferred into 14 Erlenmeyer flasks (500 mL), each containing 300 mL of liquid GYM *Streptomyces* medium. The fermentation culture was then incubated at 25 °C with 120-rpm shaking for seven days. After seven days of culture, the fermentation broth was collected and centrifuged at 3500 rpm, at 4 °C for 15 min. After removal of the pellet, sterilized XAD-7-HP resin (40 g/L) was added to the supernatant to absorb the organic products from the culture, and the resin was then shaken at 220 rpm for 4 h. The resin was filtered and de-adsorbed by a mixture of solvent acetone/MeOH (50/50, *v*/*v*). This mixture of solvent was removed under reduced pressure; the resulting aqueous layer was extracted with ethyl acetate (EtOAc; 3 × 300 mL). The EtOAc/solute extract was dried under vacuum to yield 439.5 mg of organic extract from 4.0 L of the culture.

Supplementation assays: BHA was supplemented with a quantity of 0.1 mg per 25 mL of liquid GYM *Streptomyces* medium at day zero of the culture to check the origin of isolated compounds.

### 3.4. Purification and Isolation

The organic extract (439.5 mg) from strain PC-GYM-TO, after biological assays, was subjected to flash chromatography with a 50-g SiOH Chromabond^®^ Flash column, using a sequential mixture of solvent with increasing polarity from cyclohexane to dichloromethane, EtOAc, and MeOH for 4 h to furnish 14 fractions. Guided by HPLC analysis, the first fraction containing Compound **2** (FA, 39.6 mg) and the second fraction containing Compound **1** (FB, 46.6 mg) were purified with semi-preparative HPLC (using a Prevail^®^ C_18_ column with a gradient of 0% to 100% CH_3_OH in H_2_O for 60 min, and a flow rate of 2.5 mL/min) and preparative TLC to afford Compound **1** (5.0 mg) and Compound **2** (5.9 mg), with yields of 1.25 mg/L and 1.5 mg/L, respectively.

LC–MS was applied using a Prevail^®^ C_18_ column, with a gradient of 0% to 100% CH_3_CN in H_2_O for 60 min, a flow rate of 0.8 mL/min, a sample concentration of 1 mg/mL, and an MS full range from 100–1200.

*5,5′-Thiobis(2-tert-butyl-4-methylphenol)* (Compound **1**): white amorphous powder, retention time = 35.7 min; R_f_ = 0.45 (chloroform 100%). ^1^H NMR (300 MHz, CDCl_3_) and ^13^C NMR (75 MHz, CDCl_3_) are described in Table 1. HRESIMS *m/z* 357.1983 [M − H]^−^ (calculated for C_22_H_29_O_2_S, Δ = 0 ppm).

*Octadecyl 3-(3,5-di-tert-butyl-4-hydroxyphenyl)propanoate* (Compound **2**): while solid, retention time = 26.7 min. ^1^H NMR (300 MHz, CDCl_3_) and ^13^C NMR (75 MHz, CDCl_3_) are described in Table 2. HRESIMS *m/z* 553.4592 [M + Na]^+^ (calculated for C_35_H_32_O_3_Na).

### 3.5. Molecular Models and Dynamic Simulations

The structures of Compound **1** and Santonox were built using the Yasara program and were parameterized for the Yamber3 force field following the automated AutoSMILE procedure [20]. Both geometries were optimized through the standardized minimization protocol of Yasara. Finally, to enhance the conformational space exploration available to the structures, molecular models were used as an initial point for molecular dynamics (MD) simulations. Each isomer was placed in an explicit chloroform solvent box and simulated under periodic boundary conditions at a constant temperature of 300 K. Structures were relaxed during a 2-ns MD simulation and trajectories were collected at 1-ps intervals. Analyses of the MD trajectories (root-mean-square deviation (RMSD) and clustering) was performed using Gromacs tools [21].

### 3.6. Cytotoxicity Assays

The cytotoxic assays were performed on pure compounds (with a concentration for each sample as 40 mg/mL) against HaCaT human keratinocytes and B16 murine melanoma cell lines as described in the literature [19]. HaCaT (2000 cells/well) and B16 (1800 cells/well) were cultivated in Roswell Park Memorial Institute RPMI 1640 medium supplemented with 5% fetal calf serum (FCS) and antibiotics in an atmosphere of 5% CO_2_ at 37 °C. After a 24-h culture, the samples were added at different concentrations (1, 10, 50, 100, and 200 µg/mL) and each 96-well plate was continuously incubated at the same temperature and atmosphere as above. After a 48-h culture, cell growth and viability were then measured at 540 nm using a 3-(4,5-dimethylthiazol-2-yl)-2,5-diphenyltetrazolium bromide (MTT) assay. Doxorubicin was used as a positive control. Each experiment was repeated three times.

### 3.7. DNA Damage Assays

U2OS cells were cultivated in Dulbecco's Modified Eagle's medium DMEM supplemented with 10% fetal calf serum and antibiotics in an atmosphere of 5% CO_2_ at 37 °C. The γH2AX staining was performed as previously described [22]. Images were acquired on an ArrayScan VTI high-content screening reader with a 320 lens (Thermo Scientific, Villebon sur Yvette, France). The images were analyzed using the Cell Profiler software (http://www.cellprofiler.org, Broad Institute). For all analyses, raw data files were obtained with the total amount of Hoechst fluorescence and the total amount of γH2AX fluorescence. The number of γH2AX foci per nucleus is indicated for each condition in Table 4 with more than 3000 cells counted except for the 1 µM concentration because of the high cytotoxicity.

## 4. Conclusions

In summary, two *tert*-butylphenol compounds were firstly isolated from the culture of a bacterium, *P. odorifer*, associated with the lichen, *Rhizocarpon geographicum.* Compound **1** displayed a symmetric structure including two units of BHA linked by a sulfur bond. This point can be explained by the fact that Compound **1** was putatively formed via the bioaccumulation of BHA from the culture flask used in the culture process, followed by the biotransformation of BHA into Compound **1**. Therefore, a putative biosynthesis pathway was proposed for this compound, and involved an iron–sulfur cluster protein with cysteine as a sulfur donor. Compound **1** exhibited a moderate cytotoxicity, making it promising for further investigation to determine its mechanism. The results also highlighted *P. odorifer* as a new case of *tert*-butylphenol-utilizing bacterium.

## Figures and Tables

**Table 1:** One-dimensional (1D) and two-dimensional (2D) NMR data for Compound **1** and standard Santonox in CDCl_3_ (300 MHz for ^1^H-NMR, and 75 MHz for ^13^C-NMR).

Compound 1							Standard Santonox				
Position	δ_C_, ppm	δ_H_, (ppm) mult. (*J* in Hz)	HMBC (H → C)	COSY	NOESY	Position	δ_C_, ppm	δ_H_, (ppm) mult. (*J* in Hz)	HMBC (H → C)	COSY	NOESY
1/1′	153.4	-	-	-		1/1′	153.2	-	-	-	-
2/2′	134.6	-	-	-		2/2′	134.5	-	-	-	-
3/3′	130.8	7.00, s	1, 4, 5, 8	7, 9/10/11	9/10/11	3/3′	130.6	7.00, s	1, 4, 5, 6, 7, 8	7, 9/10/11	9/10/11
4/4′	125.5	-	-	-		4/4′	137.7	-	-	-	-
5/5′	137.8	-	-	-		5/5′	125.4	-	-	-	-
6/6′	118.8	6.56, s	1, 2, 4, 7	7		6/6′	118.7	6.54, s	1, 4, 5, 7, 8	7	7
7/7′	19.8	2.28, s	4, 5, 6	3, 6		7/7′	19.7	2.27, s	4, 5, 6	3, 6	6
8/8′	34.4	-		-		8/8′	34.3	-	-	-	
9, 10, 11 /9′,10, 11′	29.7	1.28, s	9/10/11, 2, 8	3	3	9, 10, 11/9′,10, 11′	29.5	1.28, s	9/10/11, 2, 8	3	3
OH		4.72, br	-			OH					

**Table 2:** 1D and 2D NMR data for Compound **2** in CDCl_3_ (300 MHz for ^1^H-NMR, and 75 MHz for ^13^C-NMR).

Compound 2					
Position	δ_C_	Type	δ_H_, mult. (*J* in Hz)	COSY	HMBC (H → C)
1′	152.1	C	-	-	
2′	135.8	C	-	-	
3′	124.8	CH	6.99, s		1′, 5′, 12′
4′	131.1	C	-		
5′	124.8	CH	6.99, s		1′, 3′, 1, 8′
6′	135.8	C	-		
1	31.0	CH_2_	2.85, dd (9.1, 6.9)	2	3′/5′, 4′, 2, 3
2	36.5	CH_2_	2.60, dd (9.1, 6.9)	1	5′, 1, 3
3	173.4	C	-		
1″	64.6	CH_2_	4.07, t (6.8)	2″	3, 2″, 3″
2″	29.7	CH_2_	1.56–1.61, m	1″, 3″	1″, 3″
3″	29.7	CH_2_	1.56–1.61,m	2″	2″
4″–17″	22.7–32.0	CH_2_	1.24, m	18″	5″–17″, 2″, 3″, 18″
18″	14.1	CH_3_	0.88, t (6.7)	17″	17″
7′/11′^c^	34.3	C	-		
8′,9′,10′/12′,13′,14′ ^a^	30.3	CH_3_	1.43, s		8′,9′,10′/12′,13′,14′, 7′/11′, 2′, 6′, 3′/5′
OH			5.07, bs		1′, 2′, 6′

^a^ Carbons 7′/11′ and 8′,9′,10′/12′,13′,14′ form a single peak each.

**Table 3:** Cytotoxic assay of Compounds **1** and **2**.

Compound	IC_50_ (µM)	
	HaCaT	B16
**1**	8.38	4.75
**2**	>377.4	169.8 ± 1
**Doxorubicin**	0.096 ± 0.009	0.034 ± 0.001

**Table 4:** DNA damage assay of Compound **1**.

Concentration (µM)	γH2AX Foci/Nuclei
**0**	12.9 ± 0.4
**0.1**	12.8 ± 0.2
1	3.6 ± 0.3

## Supplementary Materials

Supplementary Materials are available online.
