# Supplementary material for: tert-Butylphenolic Derivatives from Paenibacillus odorifer—A Case of Bioconversion
Source: Molecules. 2018 Aug 5;23(8):1951. doi: 10.3390/molecules23081951 (PMC6222599; doi:10.3390/molecules23081951)
Supplement: Supplementary file 1 [file molecules-23-01951-s001.pdf]

Supporting information

tert-Butylphenolic Derivatives from *Paenibacillus odorifer*—A Case of Bioconversion

Thi-Bach-Le Nguyen [1], Olivier Delalande [2], Isabelle Rouaud [1], Solenn Ferron [1], Laura Chaillot [3], Rémy Pedeux [3] and Sophie Tomasi [1,*]

[1] University of Rennes 1, CNRS, ISCR—UMR 6226, F-35000 Rennes, France; nguyen.bachle@yahoo.com (T.-B.-L.N.); isabelle.rouaud@univ-rennes1.fr (I.R.); solenn.ferron@univ-rennes1.fr (S.F.)

[2] University of Rennes 1, CNRS, IGDR—UMR 6290, F-35000 Rennes, France; olivier.delalande@univ-rennes1.fr

[3] Chemistry, Oncogenesis, Stress, Signaling, Centre Eugène Marquis, Université de Rennes 1, INSERM U1242, 35000 Rennes, France; laura.chaillot@univ-rennes1.fr (L.C.); remy.pedeux@univ-rennes1.fr (R.P.)

* Correspondence: sophie.tomasi@univ-rennes1.fr; Tel.: +33-223-234-817

Table of contents | **Page**

S1. ^{1}H-NMR spectrum of compound **1** in $CDCl_3$ (300 MHz)	3
S2. Jmod spectrum of compound **1** in $CDCl_3$ (75 MHz)	3
S3. 2D-NMR HSQCedit spectrum of compound **1** in $CDCl_3$ (300 MHz)	4
S4. 2D-NMR HMBC spectrum of compound **1** in $CDCl_3$ (300 MHz)	5
S5. 2D-NMR COSY spectrum of compound **1** in $CDCl_3$ (300 MHz)	5
S6. 2D-NMR NOESY spectrum of compound **1** in $CDCl_3$ (300 MHz)	6
S7. ^{1}H-NMR spectrum of Santonox in $CDCl_3$ (300 MHz)	7
S8. Jmod spectrum of Santonox in $CDCl_3$ (75 MHz)	7
S9. 2D-NMR HSQCedit spectrum of Santonox in $CDCl_3$ (300 MHz)	8
S10. 2D-NMR HSQCedit spectrum of Santonox in $CDCl_3$ (300 MHz)	8
S11. 2D-NMR COSY spectrum of Santonox in $CDCl_3$ (300 MHz)	9
S12. 2D-NMR NOESY spectrum of Santonox in $CDCl_3$ (300 MHz)	9
S13. NOESY spectrum of fraction **1'** from the extract of the culture supplemented with BHA in Erlenmeyer flask	10
S14. ^{1}H – NMR spectra of fraction **1'** (mixture of compound **1** and BHA) (a); compound **1** (b) and BHA (c)	10
S15. ^{1}H-NMR spectrum of compound **2** in $CDCl_3$ (300 MHz)	11
S16. Jmod spectrum of compound **2** in $CDCl_3$ (75 MHz)	11
S17. 2D-NMR HSQCedit spectrum of compound **2** in $CDCl_3$ (300 MHz)	12
S18. 2D-NMR HMBC spectrum of compound **2** in $CDCl_3$ (300 MHz)	12
S19. 2D-NMR COSY spectrum of compound **2** in $CDCl_3$ (300 MHz)	13
Table 1 and S20. Major conformers for the compound **1** (0.34) and santonox (0.46) extracted from the molecular dynamics simulations performed in chloroform	14

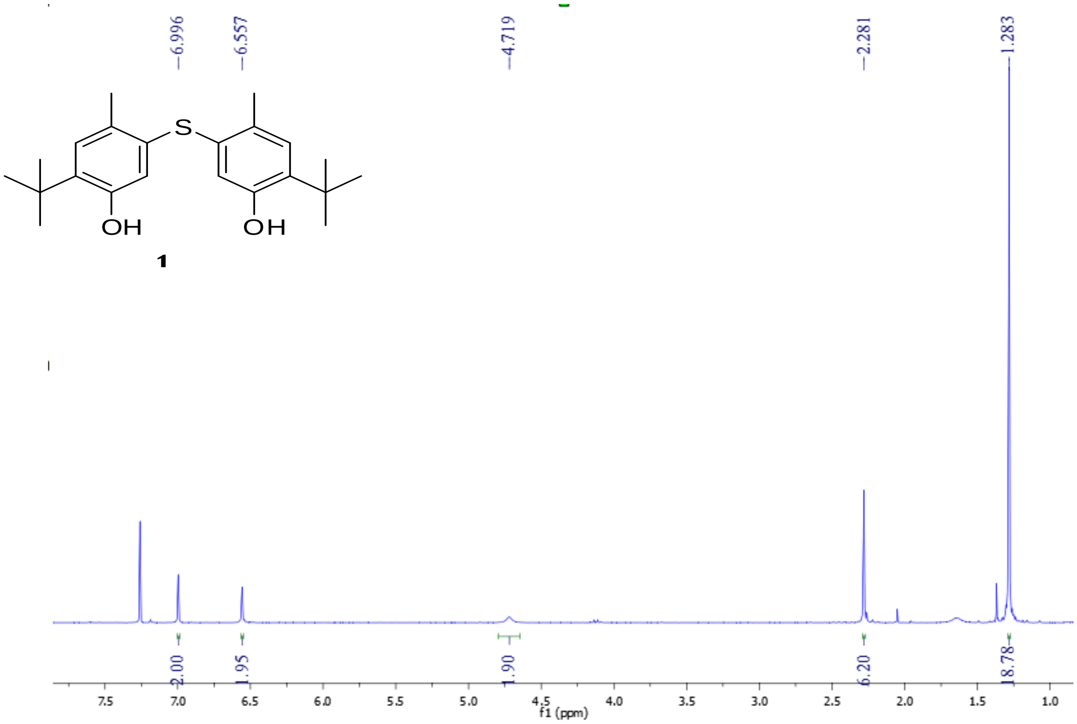

S1. ^{1}H-NMR spectrum of compound **1** in $CDCl_3$ (300 MHz)

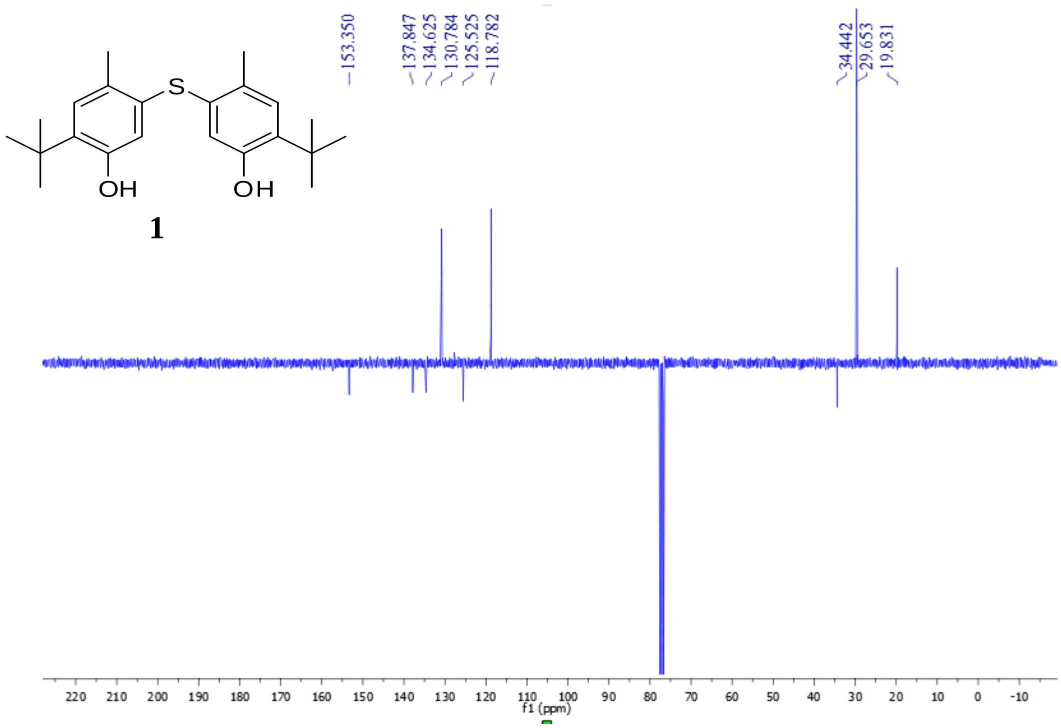

S2. Jmod spectrum of compound **1** in $CDCl_3$ (75 MHz)

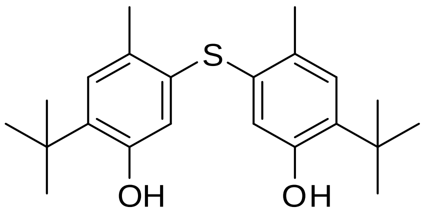

1

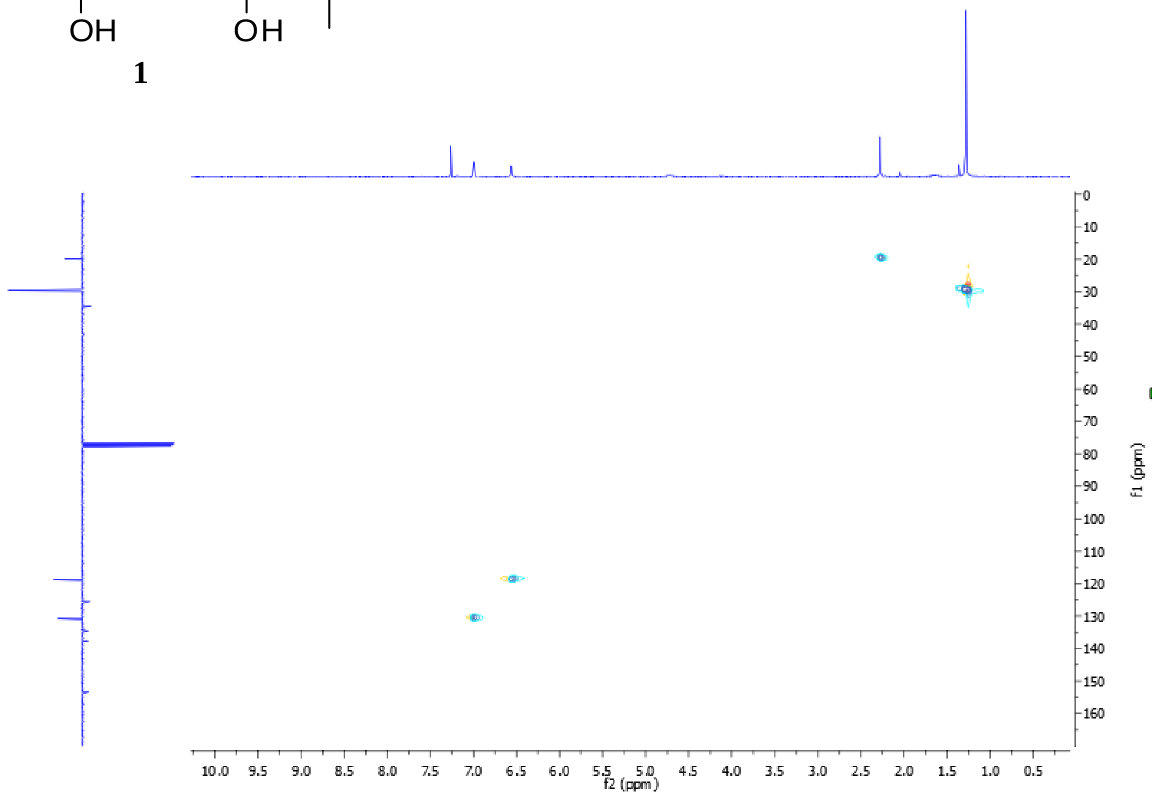

S3. 2D-NMR HSQCedit spectrum of compound **1** in $CDCl_3$ (300 MHz)

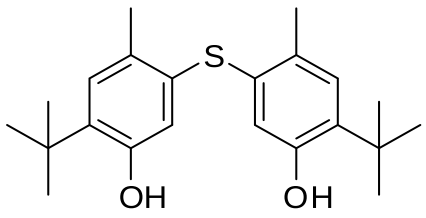

1

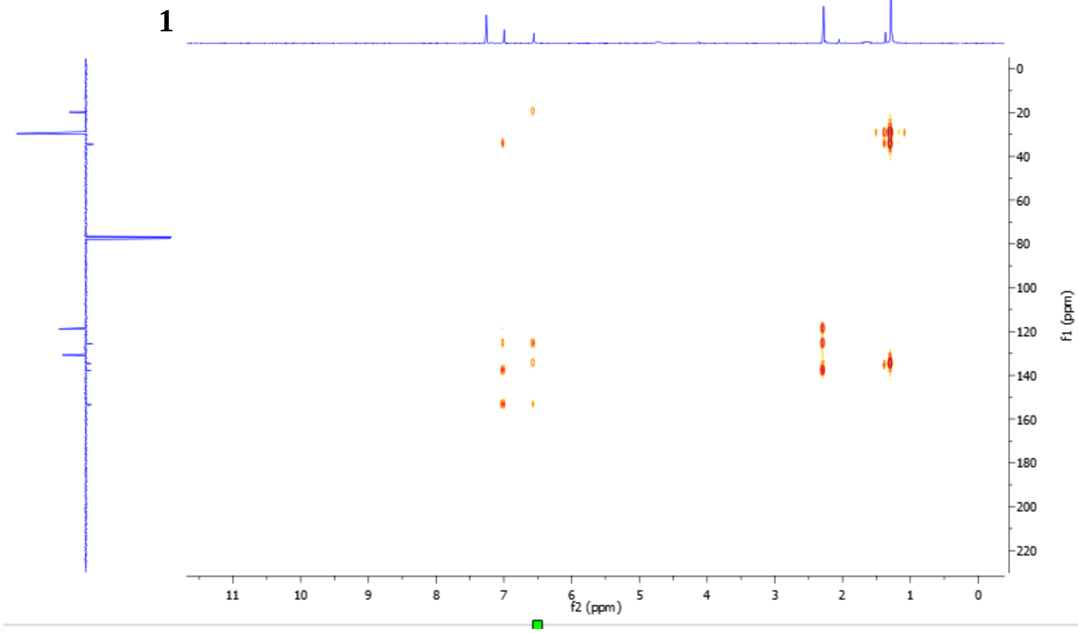

S4. 2D-NMR HMBC spectrum of compound **1** in $CDCl_3$ (300 MHz)

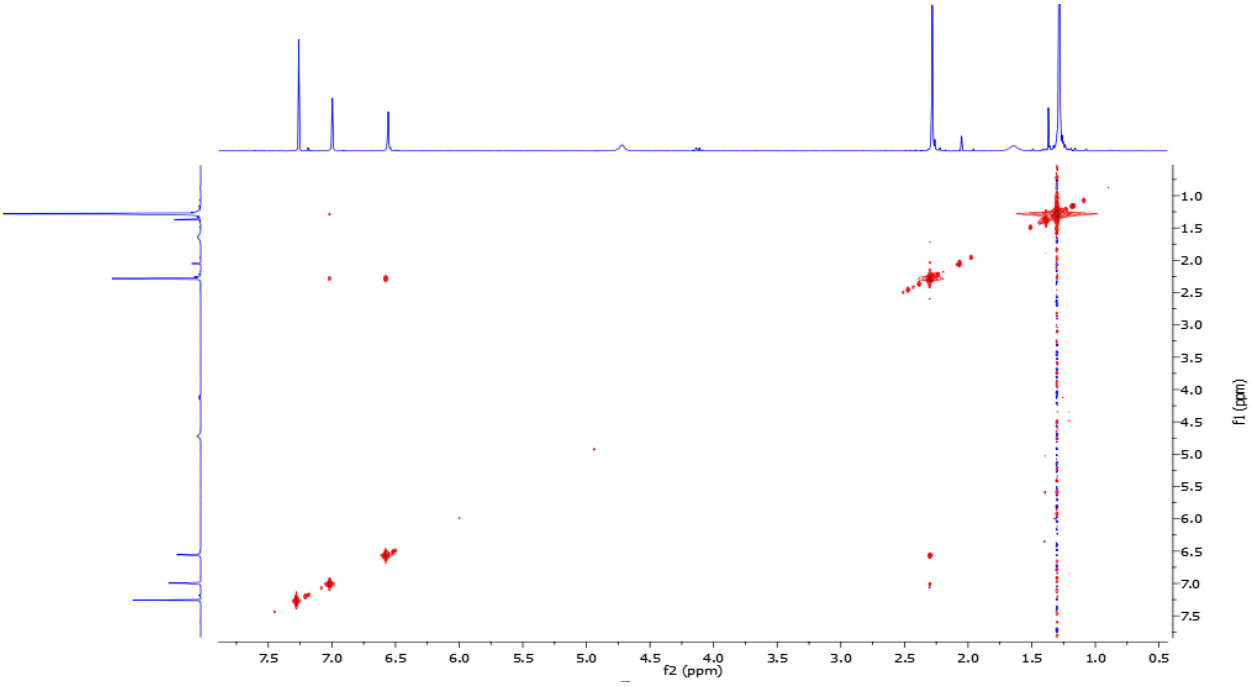

S5. 2D-NMR COSY spectrum of compound **1** in $CDCl_3$ (300 MHz)

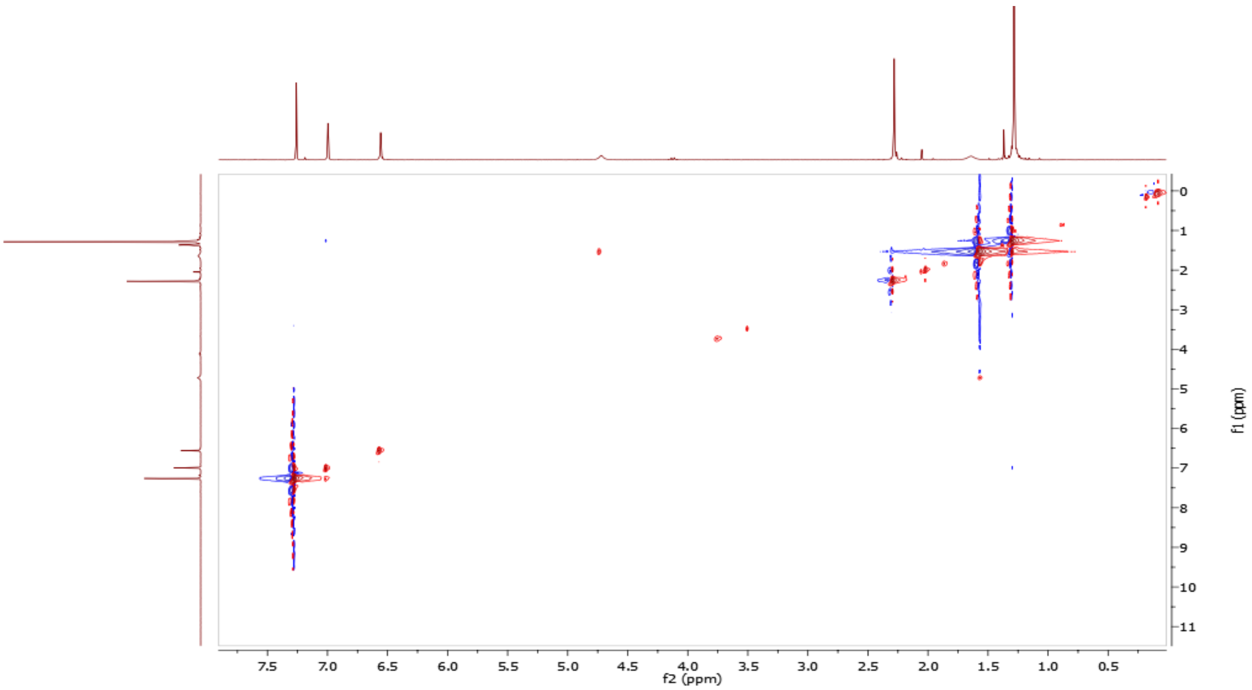

S6. 2D-NMR NOESY spectrum of compound **1** in $CDCl_3$ (300 MHz)

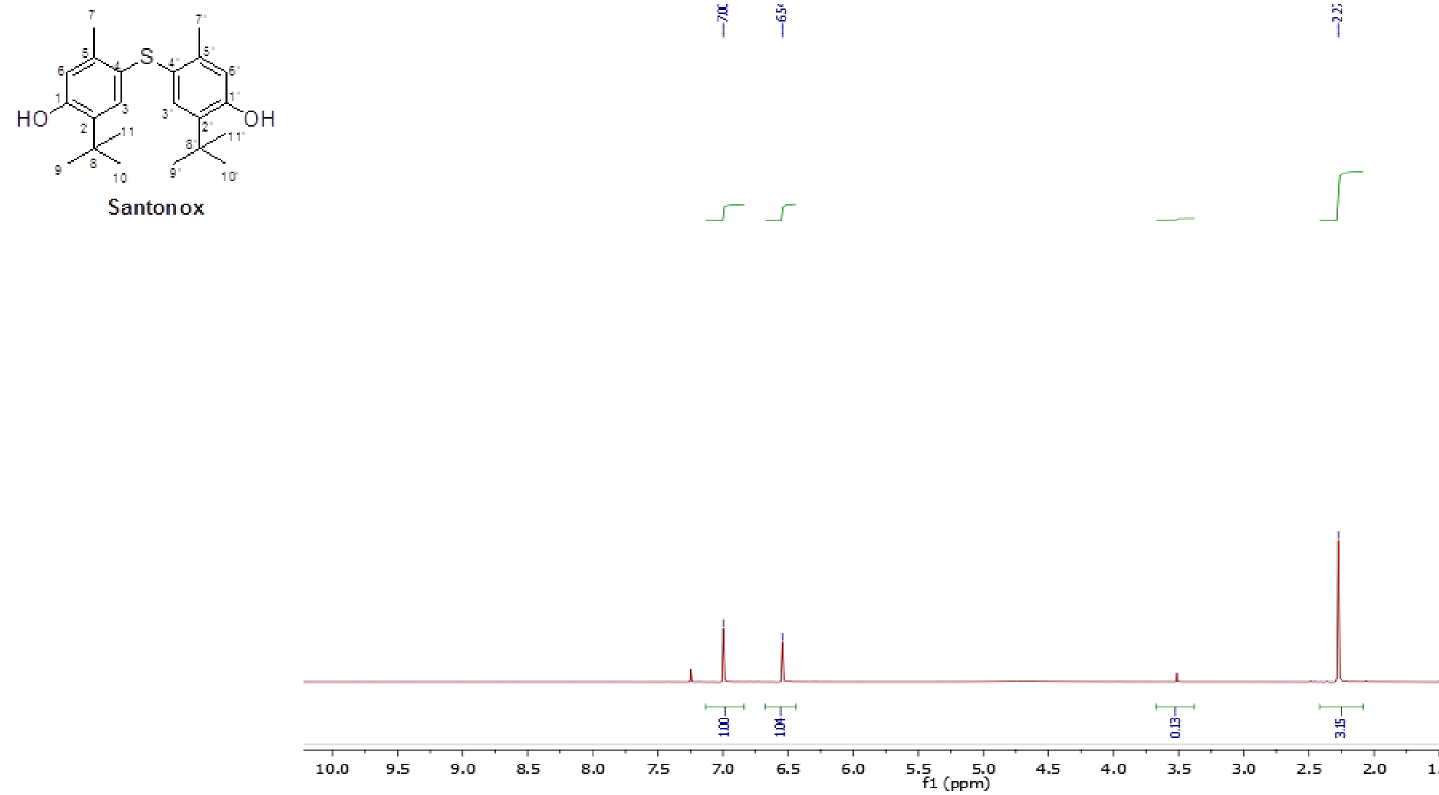

S7. ^{1}H- NMR spectrum of compound Santonox in $CDCl_3$ (300 MHz)

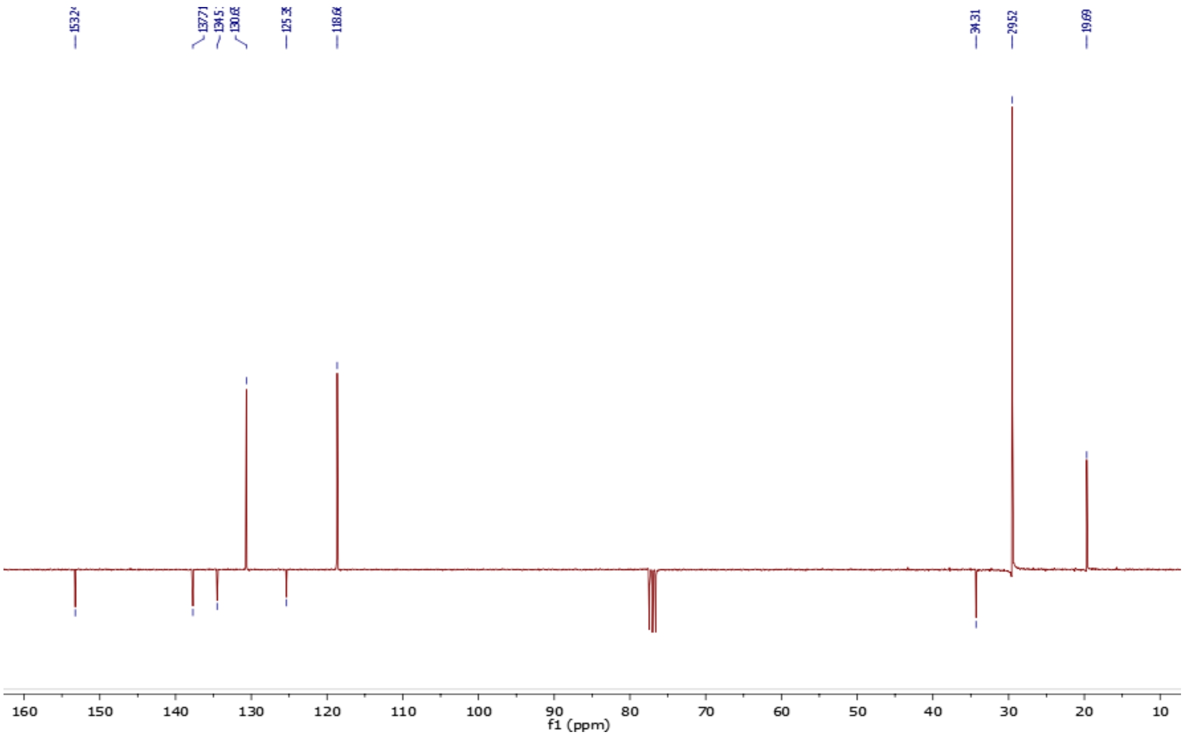

S8. Jmod-spectrum of compound Santonox in $CDCl_3$ (75 MHz)

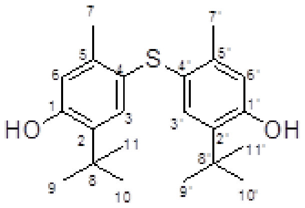

Santonox

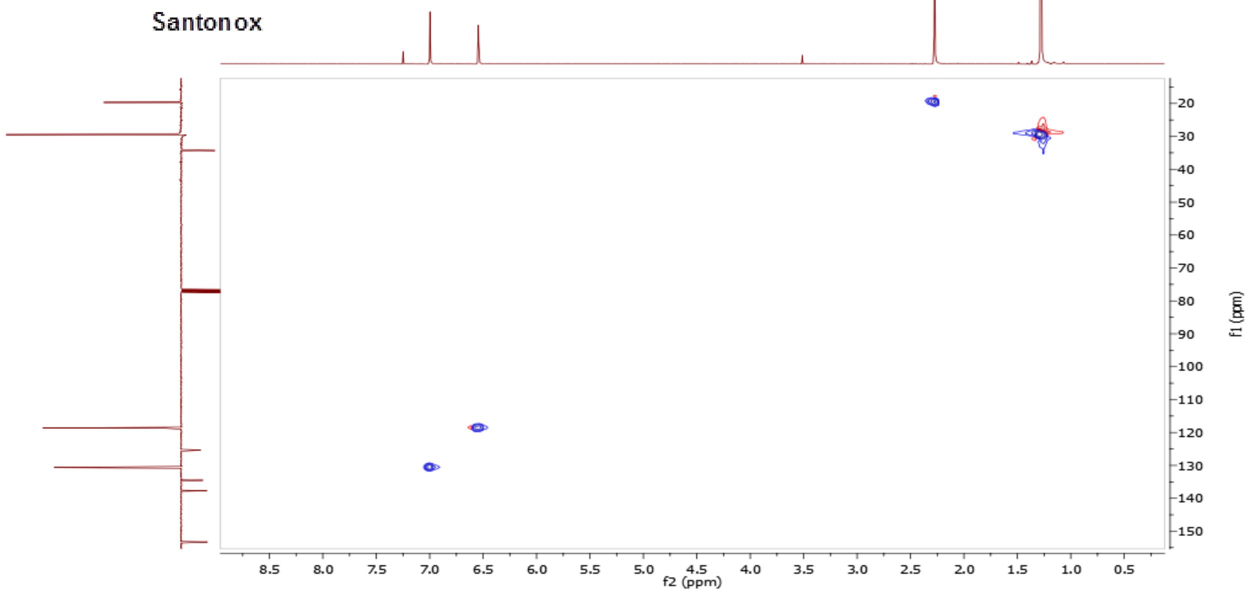

S9. 2D-NMR HSQC spectrum of Santonox in $CDCl_3$ (300 MHz)

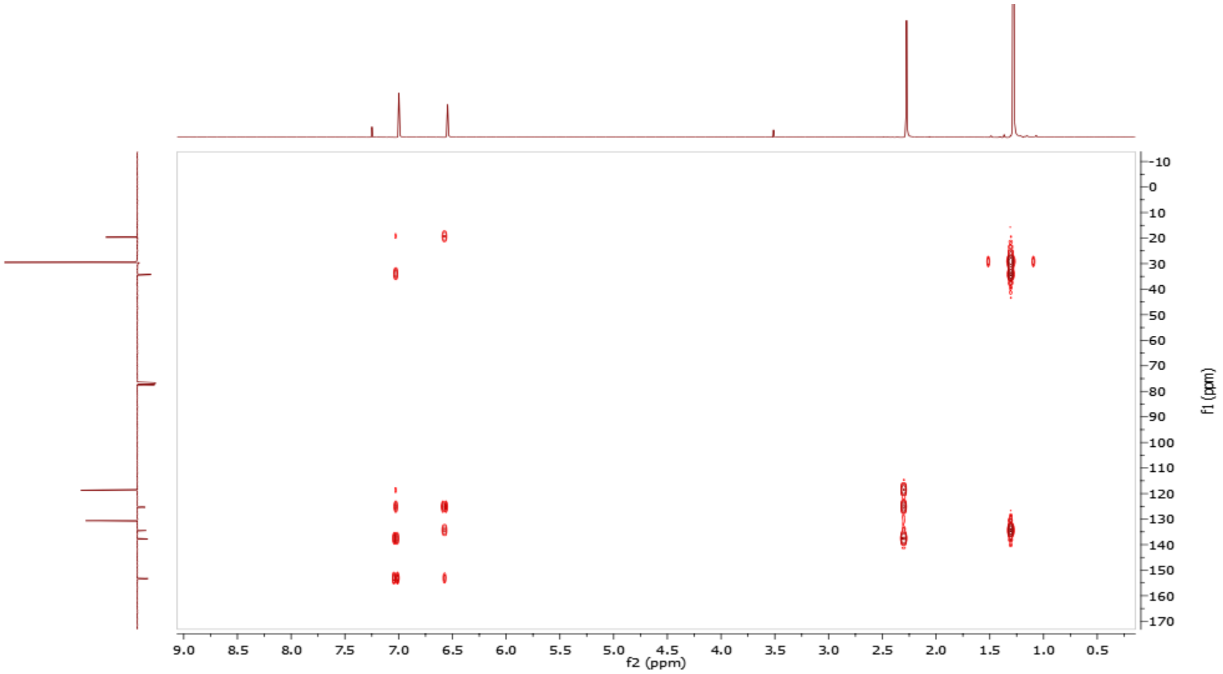

S10. 2D-NMR HMBC spectrum of Santonox in $CDCl_3$ (300 MHz)

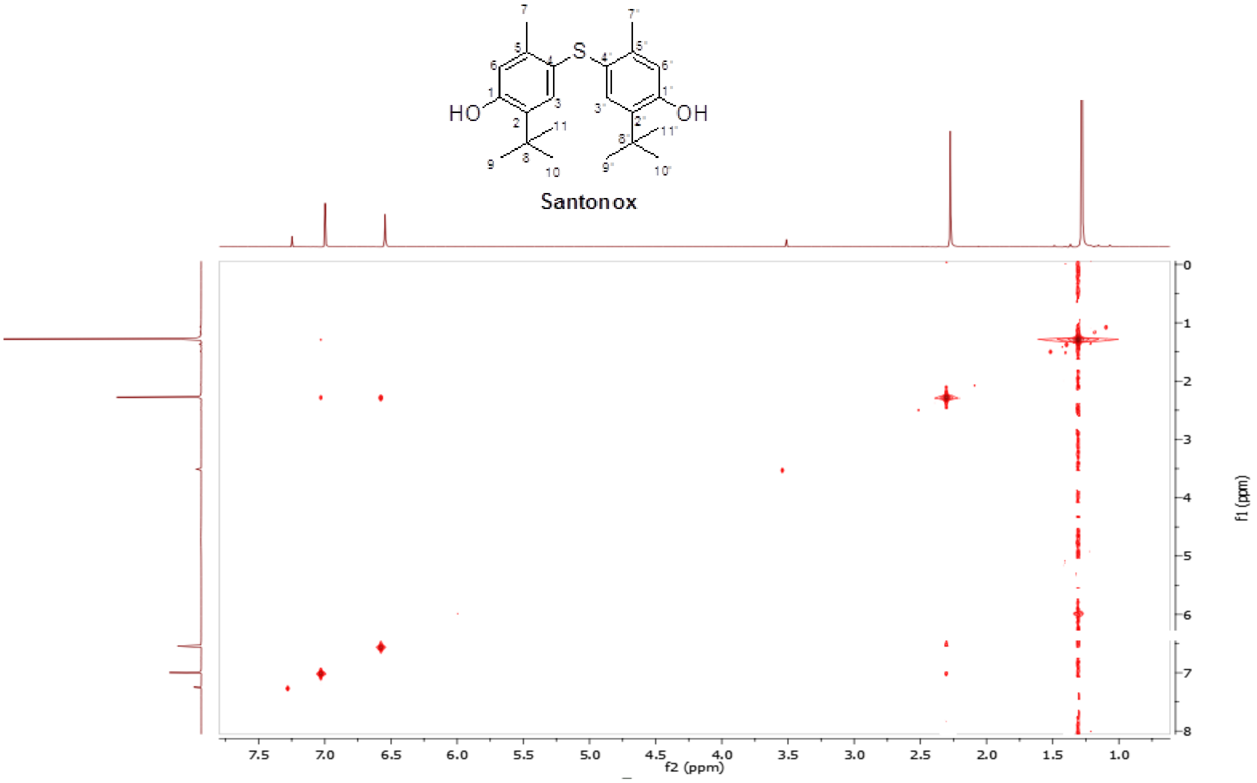

S11. 2D-NMR COSY spectrum of Santonox in $CDCl_3$ (300 MHz)

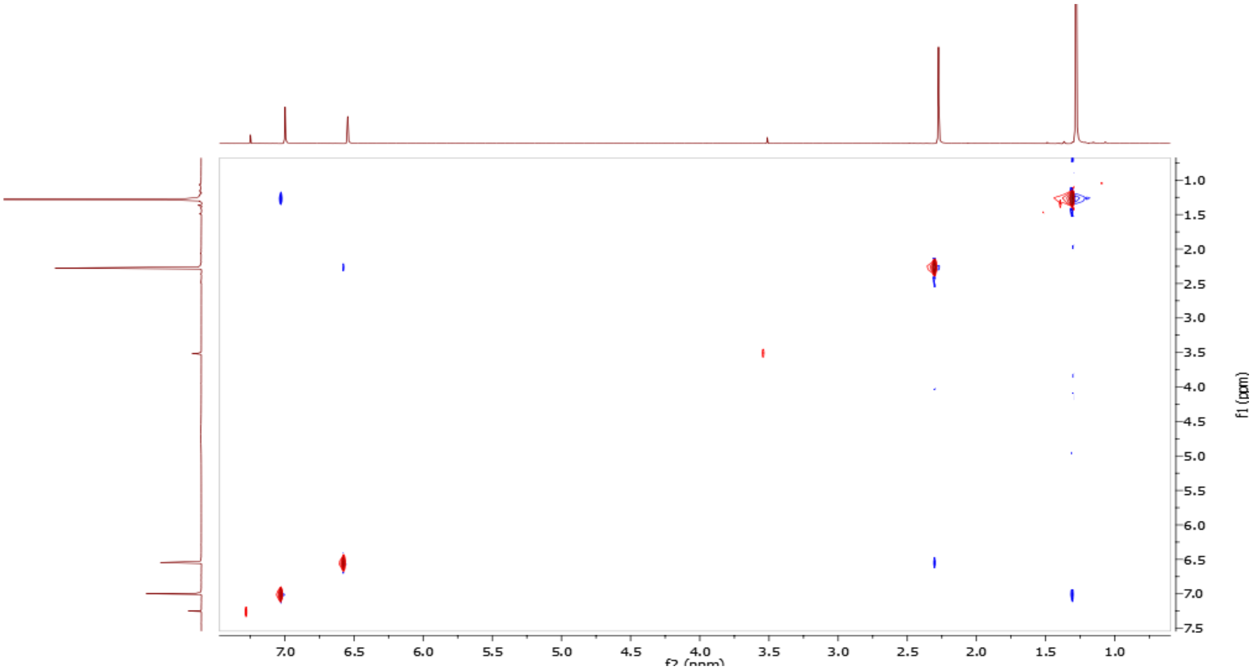

S12. 2D-NMR NOESY spectrum of Santonox in $CDCl_3$ (300 MHz)

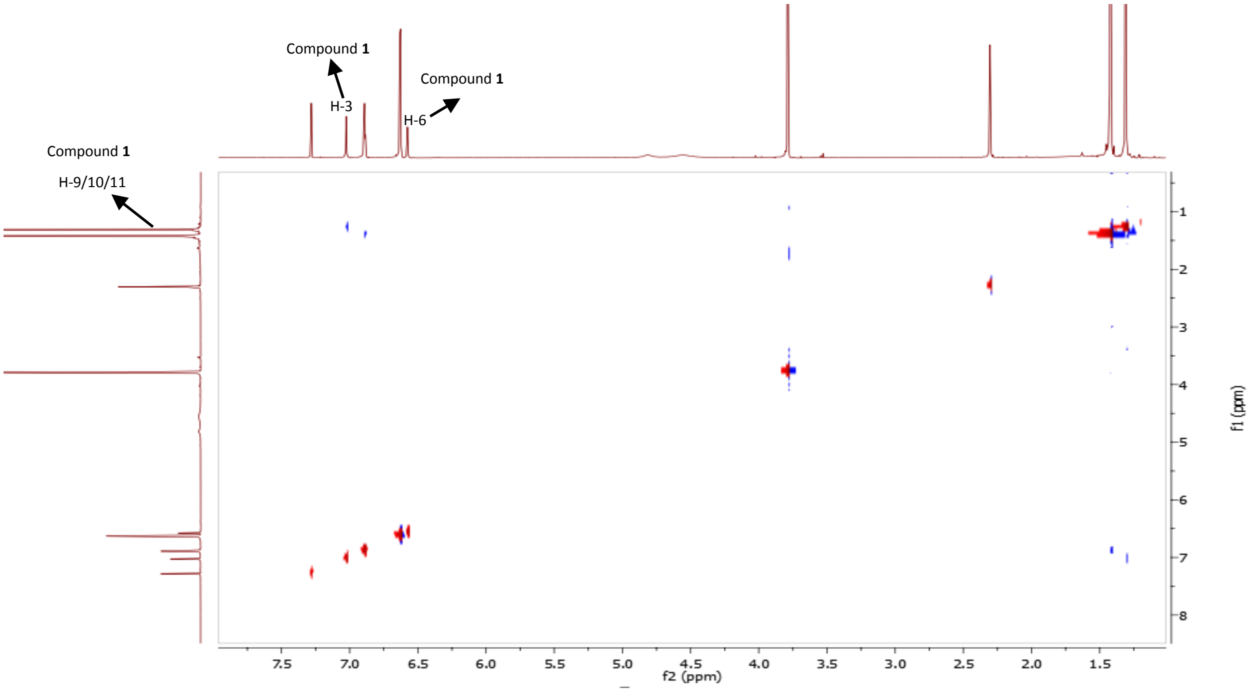

S13: NOESY spectrum of fraction **1’** (mixture of non-separable BHA and compound **1**) from the extract of the culture supplemented with BHA in Erlenmeyer flask

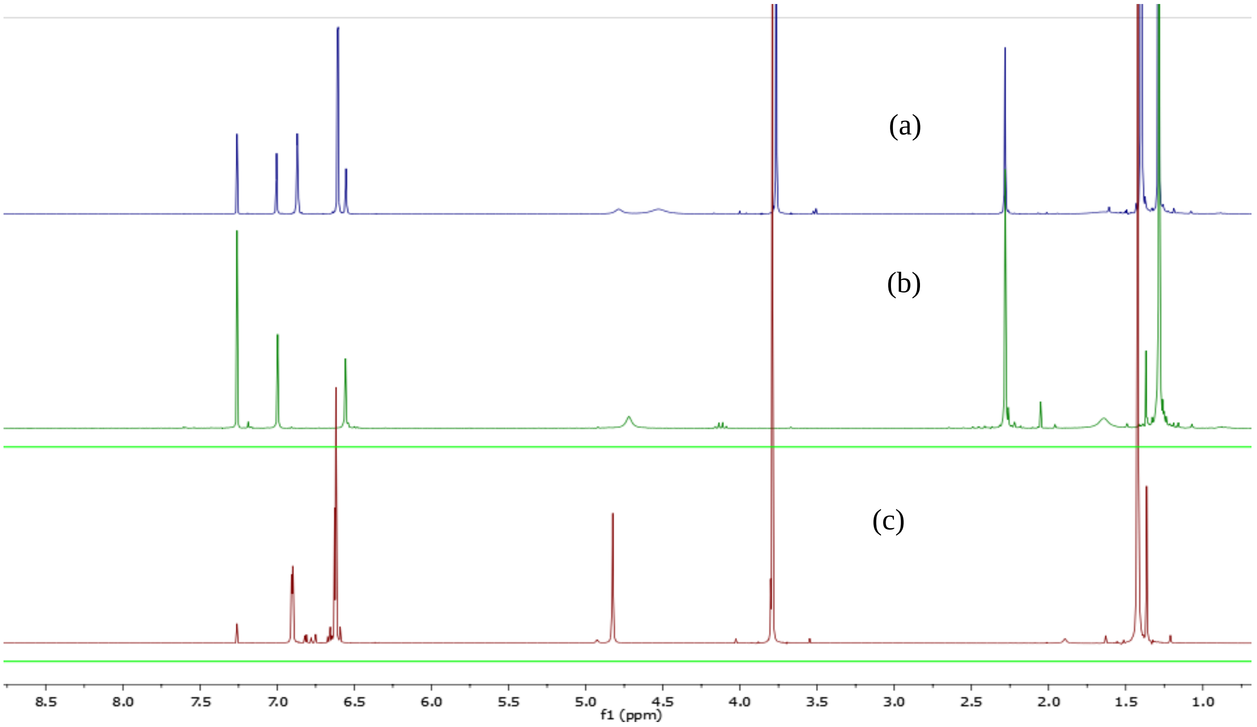

S14: ^{1}H – NMR spectra of fraction **1’** (mixture of non-separable compound **1** and BHA) (a); compound **1** (b) and BHA (c)

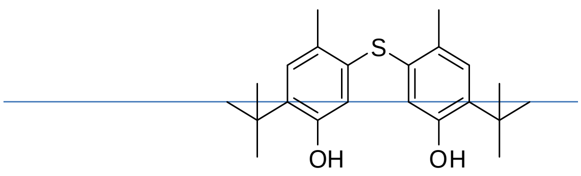

1

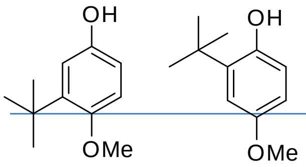

BHA

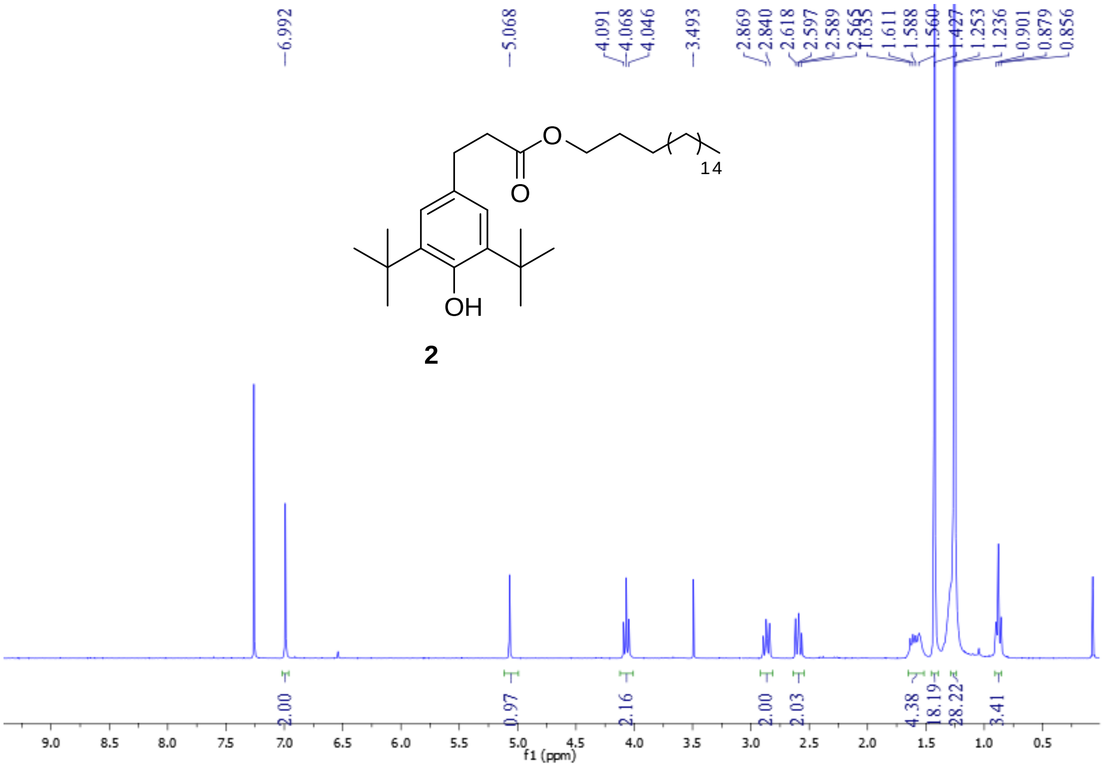

S15. ^{1}H-NMR spectrum of compound **2** in $CDCl_3$ (300 MHz)

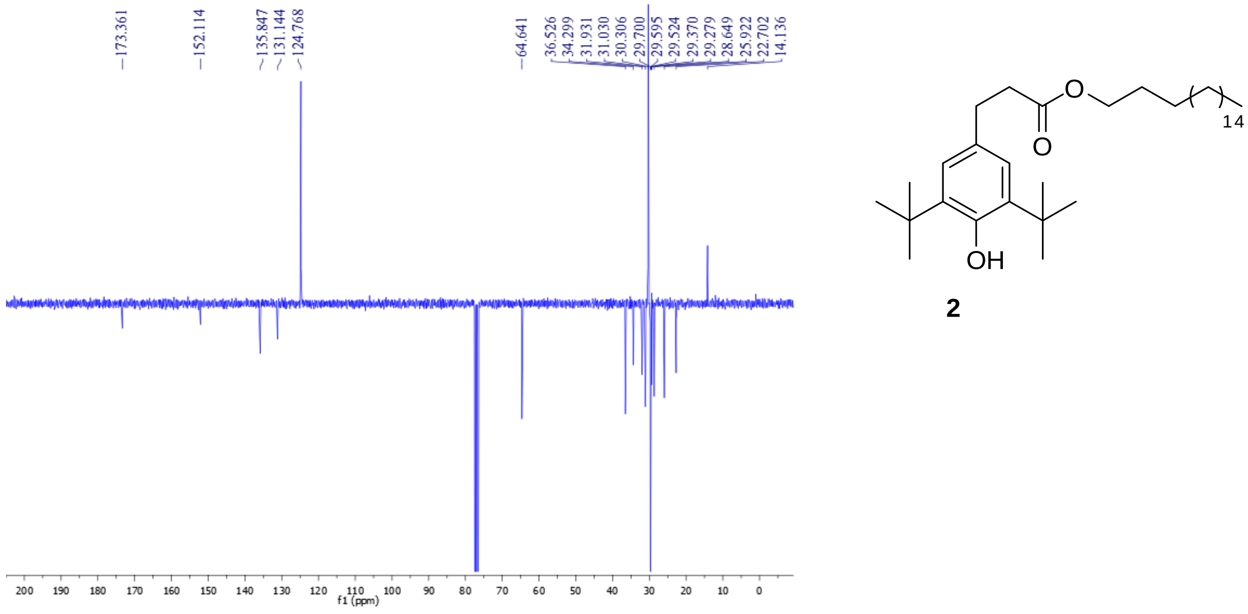

S16. Jmod-NMR spectrum of compound **2** in $CDCl_3$ (75 MHz)

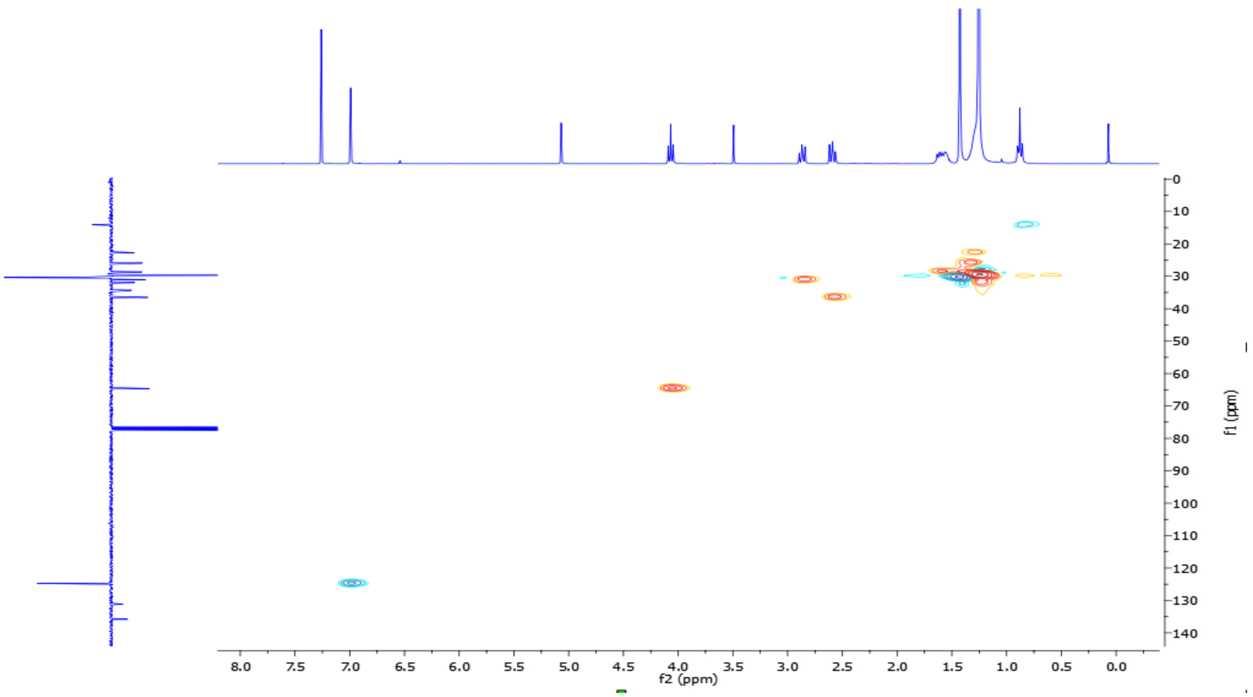

S17. 2D-NMR HSQCedit spectrum of compound **2** in $CDCl_3$ (300 MHz)

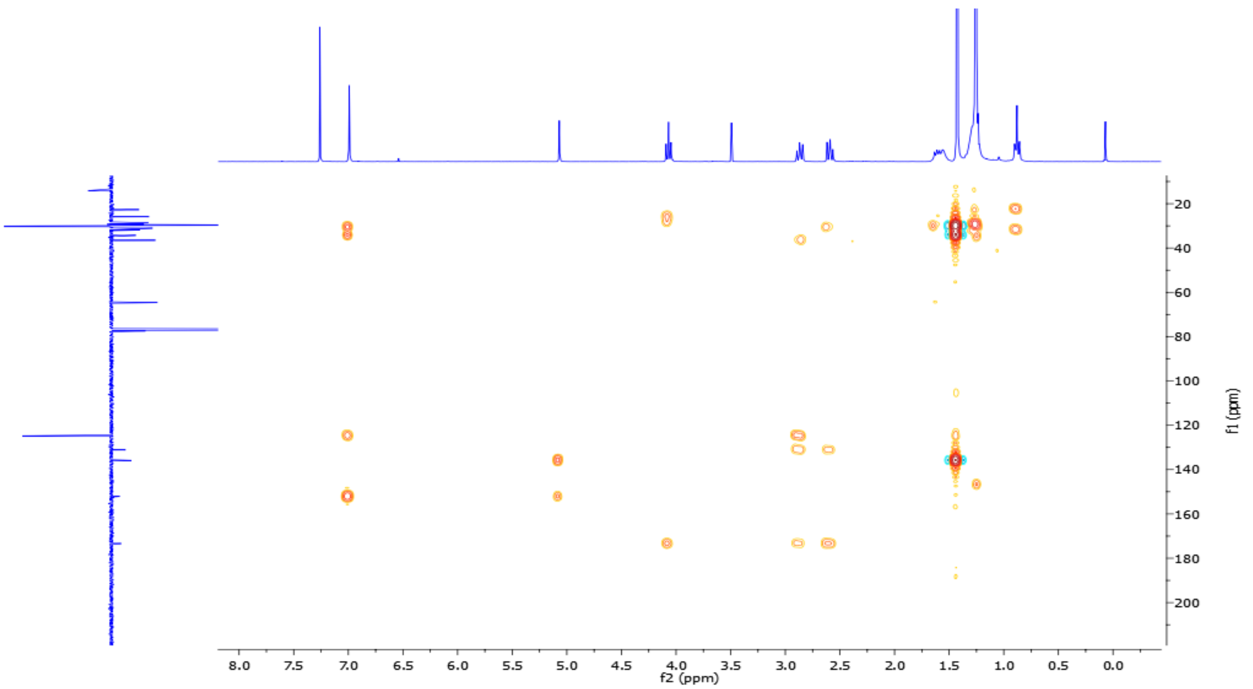

S18. 2D-NMR HMBC spectrum of compound **2** in $CDCl_3$ (300 MHz)

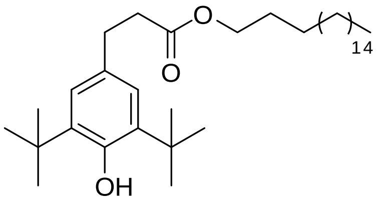

2

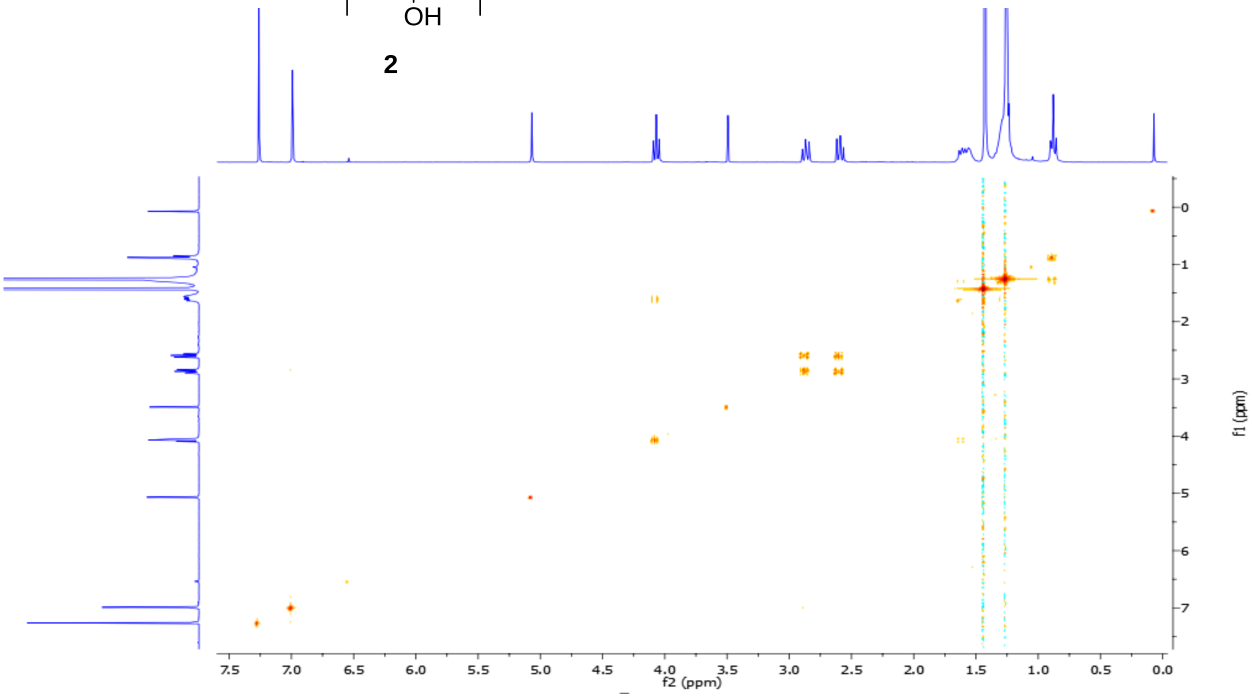

S19. 2D-NMR COSY of compound **2** in $CDCl_3$ (300 MHz)

Table S1: Predicted inter-protons distances on the base of molecular models corresponding to the major conformers of compound **1** and santonox extracted from the molecular dynamics simulation (see S20)

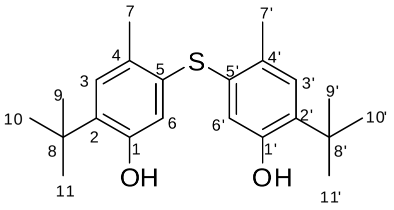

1

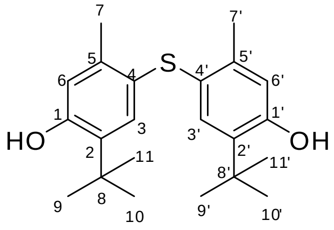

Santonox

Compound **1**		Santonox	
H3-tBut	3.87	H3-tBut	3.58
H6-tBut	5.66	H6-tBut	5.83

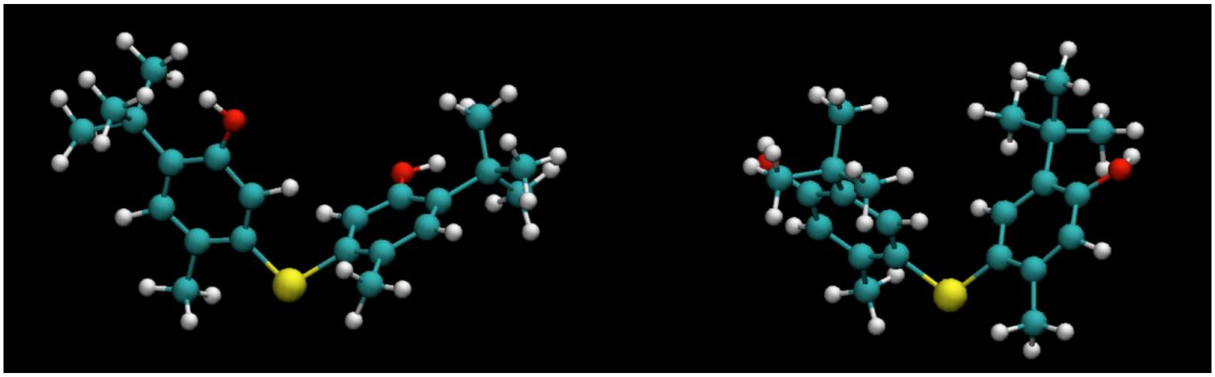

S20: Major conformers for the compound **1** (0.34) and santonox (0.46) extracted from the molecular dynamics simulations performed in chloroform.
